# Effect of pregabalin on contextual memory deficits and inflammatory state-related protein expression in streptozotocin-induced diabetic mice

**DOI:** 10.1007/s00210-016-1230-x

**Published:** 2016-03-17

**Authors:** Kinga Sałat, Joanna Gdula-Argasińska, Natalia Malikowska, Adrian Podkowa, Anna Lipkowska, Tadeusz Librowski

**Affiliations:** Faculty of Pharmacy, Department of Pharmacodynamics, Jagiellonian University Medical College, 9 Medyczna St, 30-688 Krakow, Poland; Faculty of Pharmacy, Department of Radioligands, Jagiellonian University Medical College, 9 Medyczna St, 30-688 Krakow, Poland

**Keywords:** Streptozotocin, Diabetic neuropathic pain, Pregabalin, Contextual memory, Passive avoidance task, Inflammatory state-related proteins

## Abstract

Diabetes mellitus is a metabolic disease characterized by hyperglycemia due to defects in insulin secretion or its action. Complications from long-term diabetes consist of numerous biochemical, molecular, and functional tissue alterations, including inflammation, oxidative stress, and neuropathic pain. There is also a link between diabetes mellitus and vascular dementia or Alzheimer’s disease. Hence, it is important to treat diabetic complications using drugs which do not aggravate symptoms induced by the disease itself. Pregabalin is widely used for the treatment of diabetic neuropathic pain, but little is known about its impact on cognition or inflammation-related proteins in diabetic patients. Thus, this study aimed to evaluate the effect of intraperitoneal (ip) pregabalin on contextual memory and the expression of inflammatory state-related proteins in the brains of diabetic, streptozotocin (STZ)-treated mice. STZ (200 mg/kg, ip) was used to induce diabetes mellitus. To assess the impact of pregabalin (10 mg/kg) on contextual memory, a passive avoidance task was applied. Locomotor and exploratory activities in pregabalin-treated diabetic mice were assessed by using activity cages. Using Western blot analysis, the expression of cyclooxygenase-2 (COX-2), cytosolic prostaglandin E synthase (cPGES), nuclear factor (erythroid-derived 2)-like 2 (Nrf2), nuclear factor-ĸB (NF-ĸB) p50 and p65, aryl hydrocarbon receptor (AhR), as well as glucose transporter type-4 (GLUT4) was assessed in mouse brains after pregabalin treatment. Pregabalin did not aggravate STZ-induced learning deficits in vivo or influence animals’ locomotor activity. We observed significantly lower expression of COX-2, cPGES, and NF-κB p50 subunit, and higher expression of AhR and Nrf2 in the brains of pregabalin-treated mice in comparison to STZ-treated controls, which suggested immunomodulatory and anti-inflammatory effects of pregabalin. Antioxidant properties of pregabalin in the brains of diabetic animals were also demonstrated. Pregabalin does not potentiate STZ-induced cognitive decline, and it has antioxidant, immunomodulatory, and anti-inflammatory properties in mice. These results confirm the validity of its use in diabetic patients.

**Graphical abstract:**
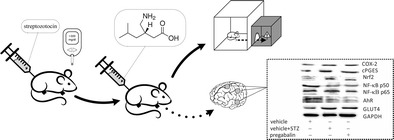
Effect of pregabalin on fear-motivated memory and markers of brain tissue inflammation in diabetic mice

Effect of pregabalin on fear-motivated memory and markers of brain tissue inflammation in diabetic mice

## Introduction

Diabetes mellitus is one of the most prevalent chronic diseases characterized by elevated blood glucose levels due to defects in insulin secretion or its action. Metabolic impairments of this disorder are a substantial cause of severe biochemical, molecular, and functional complications in many organs of the body, leading consequently to progressive damage to the whole organism (Baquer et al. 2009). The most prominent complications of long-term diabetes mellitus comprise cardiovascular diseases, stroke, chronic kidney failure, inflammation, and peripheral nerve injuries accompanied by neuropathic pain episodes (Biessels et al. 2002; Biessels and Gispen 2005; Kumar et al. 2016).

Much less recognized than diabetic neuropathic pain (DNP) and a not fully addressed complication of diabetes mellitus is cognitive dysfunction, which represents a serious medical problem in elderly patients with diabetes (Datusalia and Sharma 2014). Available studies indicate that diabetes mellitus is a risk factor for vascular type dementia (Zuloaga et al. 2015), but in recent years, a strong link has been shown between Alzheimer’s disease (AD), the most common form of dementia, and diabetes mellitus (Gasparini et al. 2002; Biessels et al. 2006; Pasquier et al. 2006; Baquer et al. 2009; Datusalia and Sharma 2014; Solmaz et al. 2015), indicating that impairments of insulin secretion or action can seriously influence not only the proper functioning of peripheral tissues, but also brain functions, being a cause of cognitive decline in diabetic patients (Pasquier et al. 2006).

The management of diabetes-induced complications comprises multiple therapeutic strategies. Among these, much attention is paid to the approaches which aim to achieve relief of DNP (Schreiber et al. 2015). This pathological condition affects more than 25 % of diabetic patients (Tesfaye et al. 2013), significantly worsening their quality of life through a negative impact on sleep, mood, and everyday functionality. In this respect, the use of analgesic adjuvants (e.g., anticonvulsant drugs, antidepressant drugs) is regarded as one of the therapeutic mainstreams in the pharmacotherapy of DNP (Finnerup et al. 2015).

One second-generation antiepileptic drug, pregabalin, is a ligand of the α_2_δ subunit of voltage-gated calcium channels which has shown high clinical efficacy in the treatment of DNP in humans (Finnerup et al. 2015; Zhang et al. 2015). It has also attenuated tactile allodynia in mouse models of neuropathic pain induced by streptozotocin (STZ) (Sałat et al. 2013a; Sałat and Sałat 2013), oxaliplatin (Sałat et al. 2014; Sałat and Sałat 2015), as well as chronic constriction injury (Sałat et al. 2015), and it is metabolically neutral (Sałat et al. 2013b), which combined validate the use of this drug as a first-line option for the treatment of DNP (Tesfaye et al. 2013).

The available literature shows that the adverse effects of pregabalin are mild, and generally, this drug is well tolerated (Rosenstock et al. 2004; Tölle et al. 2008; Moon et al. 2010; Toth 2014). The most frequently reported adverse effects of pregabalin are related to its influence on the central nervous system and these comprise dizziness, somnolence (Rosenstock et al. 2004; Toth 2014), or the propensity for abuse (Grosshans et al. 2013). Importantly, among these centrally mediated actions, little is known about the impact of pregabalin on cognition, brain glucose metabolism, or diabetes-related brain tissue inflammation. Two available research reports (Liliana et al. 2015; Javed et al. 2015) indicate that this drug may have a lower rate of cognitive side effects than other antiepileptic drugs, but these studies were not performed in diabetes-related conditions.

Hence, the present study aimed to assess the effect of pregabalin on cognition in STZ-treated mice. STZ is a nitrosourea analog antitumor drug that induces diabetes by killing β-pancreatic cells (Gao and Zheng 2014). Since the development of STZ-induced diabetes in rodents is relatively simple with a single injection, this model has been widely used to study mechanisms of diabetes, diabetic peripheral neuropathy, and DNP (Tanabe et al. 2008; Chauhan et al. 2012; Gao and Zheng 2014). Previously, it has shown that not only the intracerebral application of STZ leads to memory deficits (Blokland and Jolles 1993; Chen et al. 2013), but the intraperitoneal (ip) administration of this drug can also induce memory impairments (Cai et al. 2011; Davari et al. 2013; Miao et al. 2015; Takizawa et al. 2013). Given this fact, it is assumed that STZ can not only have a tremendous impact on sensory nerves and pain sensitivity thresholds, but it can also modulate various brain functions, including learning abilities (Davari et al. 2013). Also, biochemical and molecular alterations induced by impaired glucose metabolism can have a tremendous impact on the body’s functioning (Baquer et al. 2009; Ola et al. 2012; Coleman et al. 2015; Kumar et al. 2016), including the brain functions (Biessels et al. 2002; Gasparini et al. 2002; Baquer et al. 2009; King et al. 2015). Consequently, as a part of our research, we also evaluated the influence of STZ and pregabalin on the expression of the following proteins in the mouse brain: cyclooxygenase-2 (COX-2), cytosolic prostaglandin E_2_ synthase (cPGES), nuclear factor (erythroid-derived 2)-like 2 (Nrf2), nuclear factor-ĸB (NF-ĸB) p50 and p65, aryl hydrocarbon receptor (AhR), and glucose transporter type-4 (GLUT4).

## Materials and methods

### Chemicals

Pregabalin was purchased from Tocris Bioscience (Germany). For in vivo studies, it was dissolved in 0.9 % saline (Polfa Kutno, Poland) and administered at a dose of 10 mg/kg by the ip route 60 min before the locomotor activity test and the acquisition phase of the passive avoidance task. The dose of pregabalin tested in the present study was chosen based on the previous research (Sałat et al. 2013a) showing its significant antiallodynic activity in STZ-induced DNP in mice. STZ (Sigma-Aldrich, Poland) was dissolved in 0.1 N citrate buffer (pH 4.5) and injected in a single ip dose of 200 mg/kg (for details see the “Induction of diabetes and selection of diabetic mice for further tests” section).

### Animals and housing conditions

Adult male Albino Swiss (CD-1) mice weighing between 18 and 22 g were used in this study. The animals were housed in groups of 10 mice per cage at an ambient temperature of 22 ± 2 °C, under a light/dark (12:12) cycle. The animals had free access to food and water before experiments. The ambient temperature of the experimental room and humidity were kept constant throughout the tests. For the behavioral test, the animals were selected randomly. Each experimental group consisted of seven to eight animals. The experiments were performed between 8 AM and 2 PM. Immediately after the in vivo assay, the animals were euthanized via cervical dislocation. The procedures for animal maintenance and treatment were approved by the Local Ethics Committee of the Jagiellonian University in Krakow (ZI/862/2013).

### In vivo part

#### Induction of diabetes and selection of diabetic mice for further tests

Before the induction of diabetes, the mice were randomly divided into two groups. The first received only a vehicle (an equal volume of 0.1 N citrate buffer), while the latter was injected with ip STZ dissolved in 0.1 N citrate buffer. Blood glucose levels were measured 1 h before STZ injection (referred to as “pre-STZ”) and repeatedly 7, 14, and 21 days after STZ injection. For this purpose, a blood glucose monitoring system (AccuChek Active, Roche, France) was applied. Blood samples for the measurement of glucose concentration were obtained from the mouse tail vein. The animals were considered diabetic when their blood glucose concentration exceeded 300 mg/dl (Tanabe et al. 2008) and only those mice were used as diabetic mice in further tests.

#### Passive avoidance task

The learning abilities of diabetic mice and the effect of pregabalin on cognition were investigated using a passive avoidance task 21 days after STZ administration. For this purpose, diabetic mice were first randomly divided into pregabalin-treated or vehicle-treated groups (STZ + pregabalin-treated group and STZ + vehicle-treated group, respectively). The passive avoidance task was performed according to a method previously described by Park et al. (2012). The apparatus used in the test (Panlab Harvard Apparatus, Spain) consisted of a large white-painted illuminated compartment (26 × 26 × 34 cm) and a small black-painted dark compartment (13 × 7.5 × 7.5 cm) separated from each other by a guillotine gate. To assess the effect of pregabalin and STZ on memory impairments, the animals underwent two separate trials: an acquisition trial (conditioning phase) and a retention trial (testing phase). The latter was conducted 24 h after the acquisition trial.

In the acquisition trial, the mouse was initially placed for 30 s in the light compartment (exploration period; guillotine gate is closed). After this 30-s exploration period, the guillotine door (5 × 5 cm) between the light and the dark compartments was opened and the time elapsed before entering the black chamber was recorded. As soon as the mouse entered the dark compartment, the door was automatically closed and an electrical shock (current intensity 0.2 mA, duration 2 s) was delivered through the grid floor.

In the retention trial, the mice were placed again in the illuminated, white compartment and the latency time between door opening and entry into the dark compartment was recorded for each mouse. If the mouse did not enter the dark compartment within 180 s (cut off latency), it was concluded that it had remembered the foot shock from the acquisition trial. Better memory performance was indicated by longer latency to enter in the black chamber in the test (retention) phase than in the conditioning (acquisition) phase.

#### Activity monitoring

Activity monitoring was performed using activity cages (40 × 40 × 31 cm) supplied with I.R. horizontal and vertical beam emitters (Activity Cage 7441, Ugo Basile, Italy) connected to a counter measuring the number of light-beam interrupts. Before the test, the mice were habituated to the activity cages for a period of 30 min. In this test, 60 min before the experiment, the mice were pretreated with pregabalin. Then, the animals were placed in the activity cages in a sound-attenuated room. Software analysis enabled the measurement of the following three types of behavior during the next 30 min at 3-min intervals: ambulations, rearing, and grooming (the number of grooming bouts and the total duration of grooming activity) (Cartmell et al. 2000; Kalueff and Tuohimaa 2005).

### Ex vivo part—Western blot for quantity of protein expression

The brain tissues were collected and then homogenized on ice using T-PER (Thermo Scientific, Waltham, MA, USA) buffer with protease inhibitor cocktail set III (Calbiochem, Merck, Germany) and phosphatase inhibitors (Cayman Chemical, Ann Arbor, MI, USA). Protein concentrations were determined using the Bradford reaction. Aliquots (20 μg) were solubilized in a Laemmli buffer with 2 % mercaptoethanol (BioRad, Hercules, CA, USA) and subjected to 10 % SDS-polyacrylamide gel electrophoresis as described previously (Gdula-Argasińska et al. 2015). We used primary antibodies: anti-COX-2 (diluted 1:500), anti-cPGES (diluted 1:1000), anti-Nrf2 (diluted 1:100), anti-AhR (diluted 1:500), and anti-GAPDH (diluted 1:1000) (GeneTex Inc., Irvine, CA, USA), as well as NF-ĸB p50, NF-ĸB p65 (Cayman Chemical), diluted 1:100 and anti-GLUT4 (Sigma-Aldrich, Saint Louis, MO, USA), diluted 1:200 in Signal + for Western Blot (GeneTex). The secondary antibody was EasyBlot anti-rabbit IgG (HRP) diluted 1:1000 in Signal + for Western blot (GeneTex). Proteins were detected using a Clarity Western ECL luminol Substrate Western blotting detection kit (Bio-Rad). The integrated optical density of the bands was quantified using a ChemiDoc Camera with Image Lab software (Bio-Rad).

### Data analysis

Data analysis of the results was carried out using GraphPad Prism software (v. 5, USA). Numerical results from behavioral tests are expressed as means ± standard error of the mean (SEM). For the statistical analysis, one-way analysis of variance (ANOVA) was used, followed by Newman-Keuls post hoc comparison or two-way repeated measures ANOVA, followed by Bonferroni multiple comparison. In the ex vivo part of this research, the values are presented as means ± SD. One-way ANOVA with Tukey’s or Scheffe’s post hoc tests were performed to evaluated differences in protein expression. *P* < 0.05 was considered significant.

## Results

### Measurements of blood glucose level

**Fig. 1:**
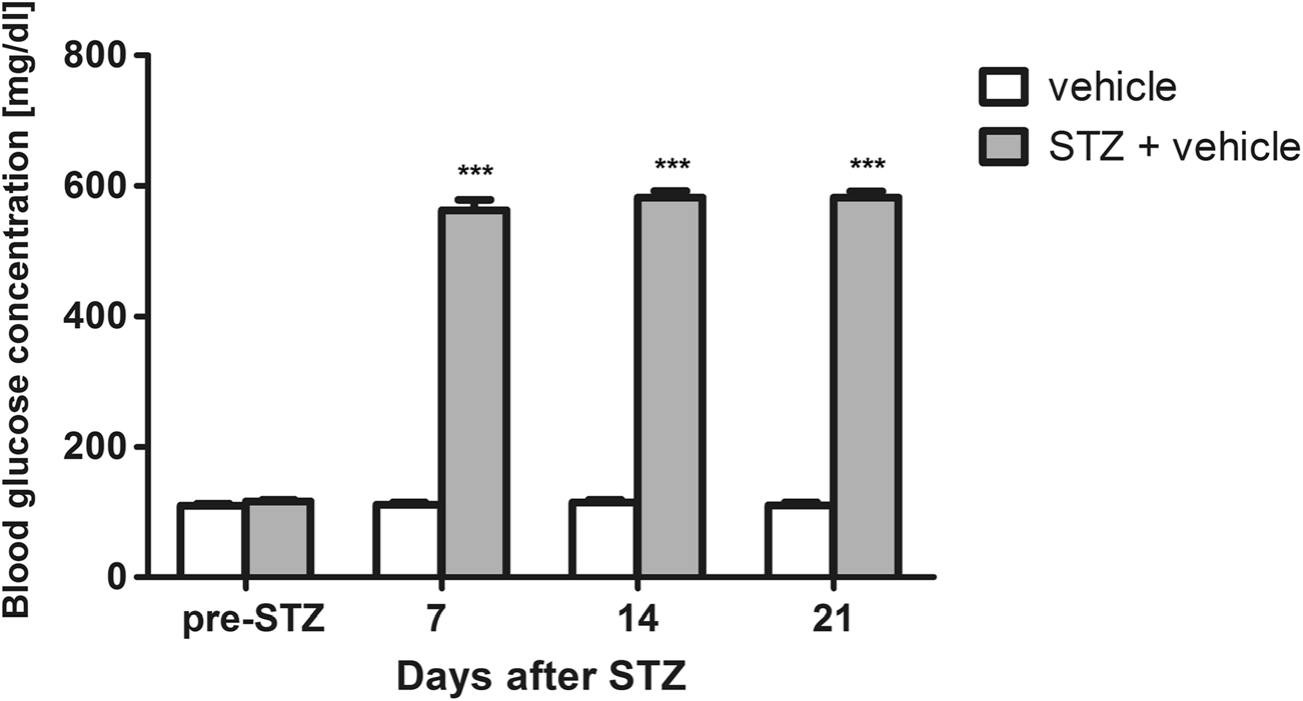
Mean blood glucose levels in vehicle-treated control mice and STZ-treated mice measured before STZ administration (pre-STZ) and then 7, 14, and 21 days after STZ injection. Statistical analysis: two-way repeated measures analysis of variance (ANOVA), followed by Bonferroni post hoc comparison. Significance vs. vehicle-treated (non-diabetic) mice: ***P < 0.001

As shown in Fig. 1, an overall effect of treatment was observed (*F*[1,66] = 956.95, *P* < 0.0001) which resulted in hyperglycemia in STZ-treated mice. Time also affected the results in a statistically significant manner (*F*[3,66] = 351.16, *P* < 0.0001). Drug × time interaction was also significant (*F*[3,66] = 343.60, *P* < 0.0001). For the first time, an elevated blood glucose level was observed 7 days after STZ administration. It was maintained during the following days (significant at *P* < 0.001 vs. normoglycemic control at each time-point). On day 21, after STZ injection, 3 h after the last measurement of blood glucose concentration, behavioral tests were performed in all experimental groups.

### Passive avoidance

**Fig. 2:**
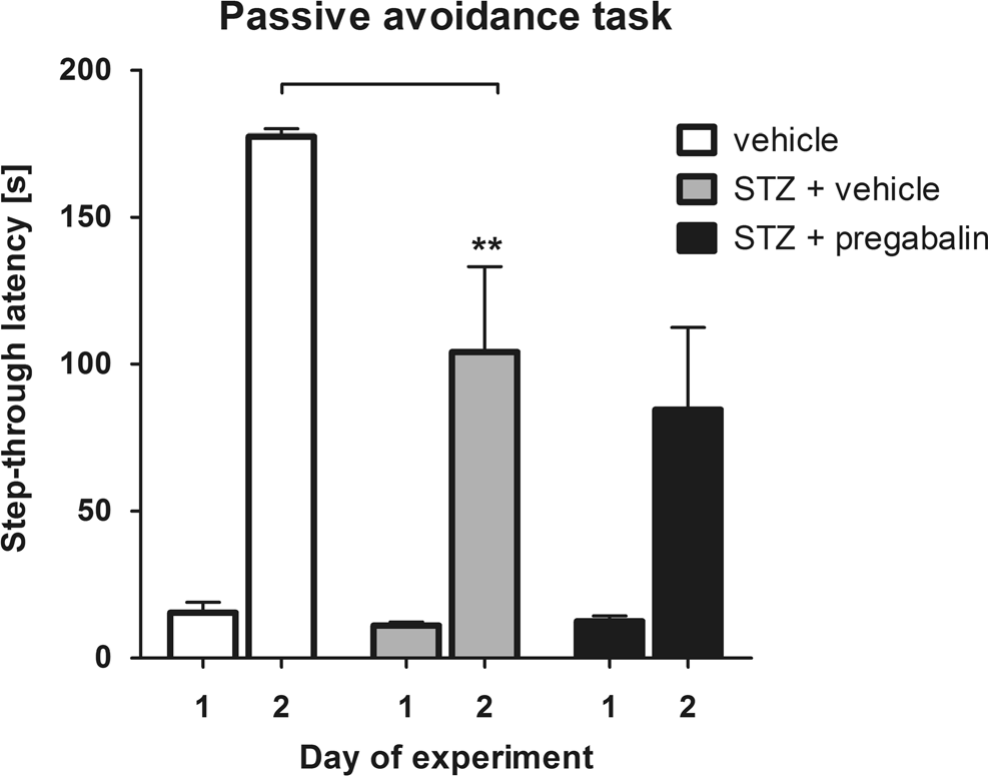
Effect of STZ and pregabalin on learning and memory in the passive avoidance task. Results are shown as the mean step-through latency (±SEM) in the acquisition phase (day 1) and in the retention phase (day 2). Statistical analysis: two-way repeated measures analysis of variance (ANOVA), followed by Bonferroni post hoc comparison. Significance vs. vehicle-treated (non-diabetic) mice in the retention phase: **P < 0.01

In this fear-motivated task, the effect of pregabalin on STZ-induced cognitive dysfunction was investigated. A significant overall effect of treatment was observed (*F*[2,19] = 5.33, *P* < 0.05). Time also affected the results significantly (*F*[1,19] = 72.86, *P* < 0.0001). Drug × time interaction was also significant (*F*[2,19] = 4.71, *P* < 0.05). In the acquisition phase, the step-through latency was similar in all experimental groups. In the retention phase of this test in all tested groups, prolongation of step-through latency was observed (Fig. 2). However, the step-through latency of STZ-treated control mice was significantly shorter compared to that of control animals not treated with STZ (*P* < 0.01). In the retention phase, the difference in the step-through latency between STZ-treated control group and STZ + pregabalin-treated mice was not statistically significant (Fig. 2).

### Activity monitoring

**Fig. 3:**
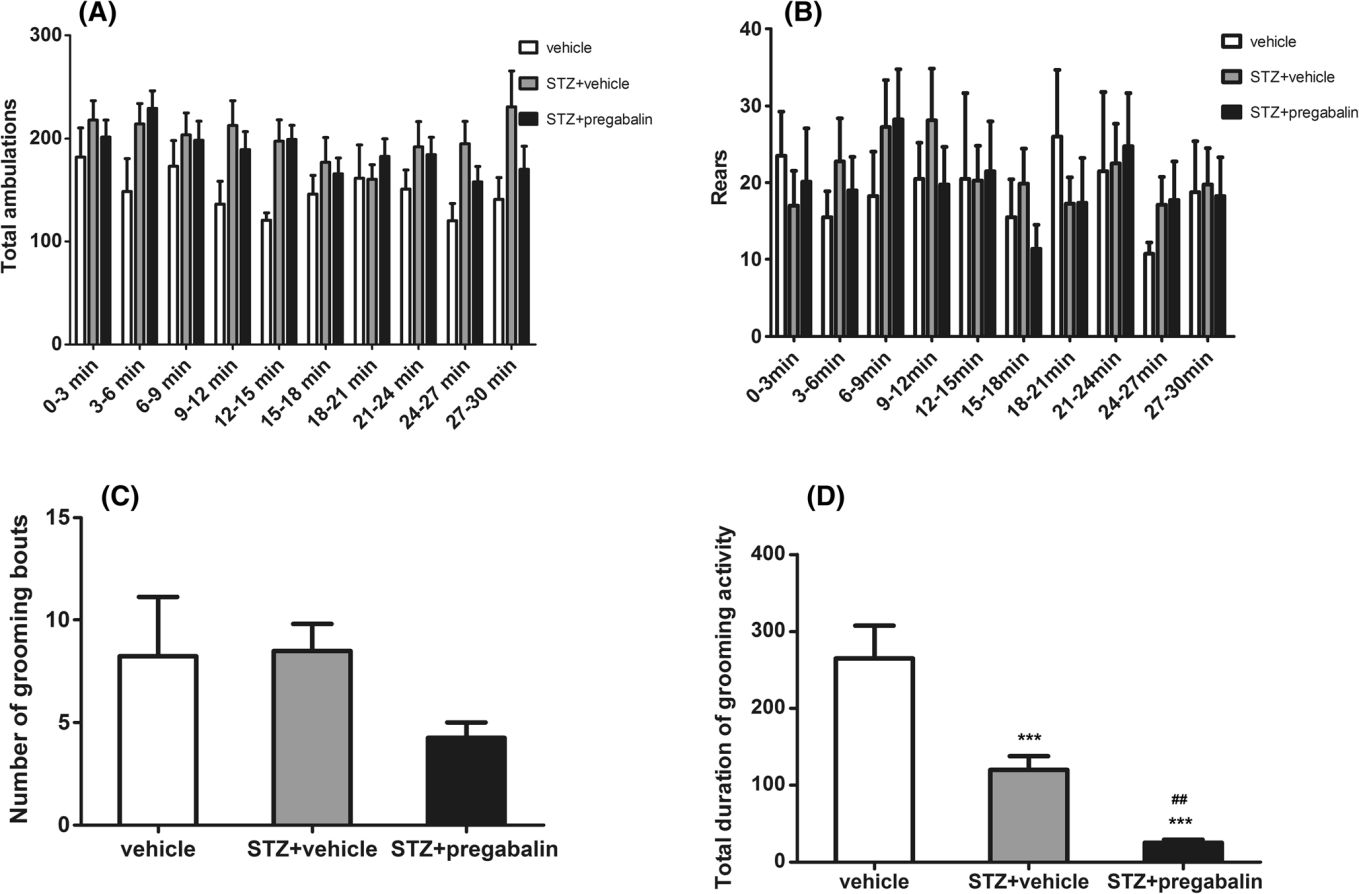
Effect of pregabalin on diabetic STZ-treated animals’ activity measured as the number of ambulations (**a**), rears (**b**), and grooming behavior (**c**, **d**). Data are expressed as mean ± SEM. Behaviors were monitored over a 30-min time period. Statistical analysis: two-way repeated measures analysis of variance (ANOVA), followed by Bonferroni post hoc comparison (**a**, **b**) or one-way ANOVA, followed by Newman-Keuls multiple comparison test (**c**, **d**). Significance vs. vehicle-treated (non-diabetic) mice: ***P < 0.001, and vs. STZ-treated control: ^##^ *P* < 0.01

Animals’ behavior was monitored by measuring ambulations, rearing, and grooming activity. None of the treatments affected the number of ambulations (*F*[2,17] = 2.37, *P* > 0.05; Fig. 3a), but time affected the results in a statistically significant manner (*F*[9,153] = 2.18, *P* < 0.05). Drug × time interaction was not significant (*F*[18,153] = 1.20, *P* > 0.05).

Treatment with STZ or pregabalin had no influence on the number of rears (*F*[2,17] = 0.05, *P* > 0.05; Fig. 3b). Time did not affect the results, either (*F*[9,153] = 1.81, *P* > 0.05). Drug × time interaction was not significant (*F*[18,153] = 0.89, *P* > 0.05).

Neither STZ, nor pregabalin affected the number of grooming bouts (*F*[2,17] = 3.137, *P* > 0.05; Fig. 3c). In contrast to this, compared to non-diabetic controls, both treatments significantly decreased the total duration of grooming behavior (*F*[2,16] = 29.16, *P* < 0.0001; Fig. 3d) by 55 and 90 %, respectively, for STZ + vehicle-treated and STZ + pregabalin-treated groups.

### Western blot for quantity of protein expression

**Fig. 4:**
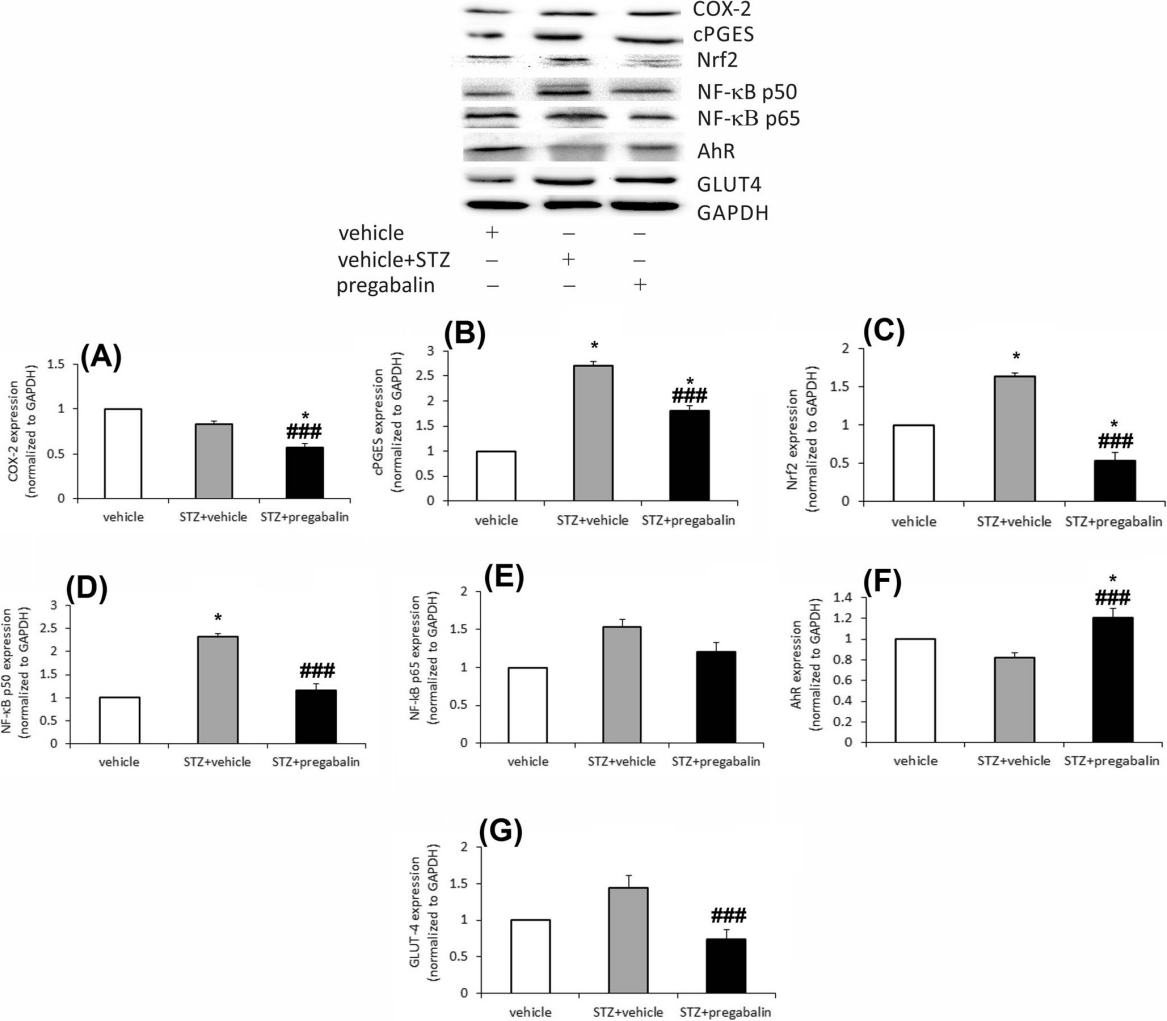
The effect of STZ and pregabalin on COX-2 (**a**), cPGES (**b**), Nrf2 (**c**), NF-ĸB p50 (**d**), NF-ĸB p65 (**e**), AhR (**f**), and GLUT4 (**g**) protein expression in mouse brains. Results are shown as fold changes of control. Statistical analysis: one-way ANOVA, followed by Tukey’s or Scheffe’s post-hoc test. Significance vs. non-diabetic vehicle-treated mice: *P < 0.05; significance vs. STZ + vehicle-treated mice: ^###^ *P* < 0.001

COX-2 expression in the brains was lower in STZ + vehicle-treated control and STZ + pregabalin-treated mice in comparison to the non-diabetic controls (*F*[2,21] = 200.17, *P* < 0.0001). Statistically significant (*P* < 0.05 vs. vehicle-treated group and *P* < 0.001 vs. STZ + vehicle-treated mice) repression of this protein was observed in diabetic mice treated with pregabalin (Fig. 4a). We observed different expression levels of cPGES in all experimental groups (*F*[2,21] = 977.86, *P* < 0.0001). Statistically, the highest expression of cPGES protein was observed in the brains of STZ + vehicle-treated mice when compared to the STZ + pregabalin-treated group (*P* < 0.001). The level of this protein was higher in the brains of those groups, when compared to vehicle-treated mice (*P* < 0.05; Fig. 4b). Also, there was a significant difference in Nrf2 expression in the three experimental groups (*F*[2,21] = 214.57, *P* < 0.0001). In the brains of pregabalin-treated diabetic animals, a statistically significant repression of Nrf2 was observed (*P* < 0.05 vs. control and *P* < 0.001 vs. STZ + vehicle; Fig. 4c). The highest amount of Nrf2 was observed in the brains of STZ + vehicle control mice, when compared to control non-diabetic mice (*P* < 0.05) and the STZ + pregabalin-treated group (*P* < 0.001). NF-ĸB p50 expression was significantly higher in the brains of STZ control animals (two-fold), when compared to STZ + pregabalin-treated mice (*P* < 0.001) and non-diabetic controls (*P* < 0.05) (*F*[2,21] = 891.39, *P* < 0.01; Fig. 4d). The expression of NF-ĸB p65 remained unchanged in the brains of STZ control and STZ + pregabalin-treated mice (Fig. 4e). Compared to the vehicle-treated group, the expression of AhR was lower in the brains of STZ controls (not significant). It was statistically higher in STZ + pregabalin-treated mice compared to the vehicle-treated group (*P* < 0.05) and STZ controls (*P* < 0.001) (*F*[2,21] = 208.22, *P* < 0.0001; Fig. 4f). After treatment with pregabalin, a significant (*P* < 0.001) repression of GLUT4 was observed in mouse brains, when compared to STZ-treated control animals (*F*[2,21] = 358.52, *P* < 0.0001; Fig. 4g).

## Discussion

Long-term diabetes mellitus is a disease that not only impairs the endocrine homeostasis of an organism, but also affects the functioning of the central and peripheral nervous systems (Biessels et al. 2002; King et al. 2015). The disease itself is regarded as one of the risk factors for AD (Nguyen et al. 2014; Ohara et al. 2011) and diabetes-induced cognitive decline, accompanied by biochemical impairments within diabetic brains, resembles that typical for AD (Court and Perry 1991; Giacobini et al. 1989; Hoyer 2002a, b; Mao et al. 2014). Hence, these cognitive deficits seem to be particularly troublesome and serious, and therefore, it is important to treat diabetic patients using drugs which do not aggravate these potential pre-existing memory deficits.

In the present study, to induce diabetes, we used STZ as a convenient and easy-to-use tool (Gao and Zheng 2014). When treated with a single dose of STZ, most mice and rats become hyperglycemic within several days, and after 2 to 3 weeks, they exhibit behavioral signs of DNP (Tanabe et al. 2008; Sałat et al. 2013a), i.e., tactile allodynia and thermal hyperalgesia in response to mechanical and thermal stimuli, respectively (Tanabe et al. 2008; Gao and Zheng 2014).

The duration of diabetes mellitus is thought to be one of the key factors for rodent studies on DNP and its effective treatment (Obrosova 2009), as well as diabetes-related cognitive deficits (Biessels and Gispen 2005). This is due to the fact that both these diabetes-related complications share a common etiology, i.e., neuropathy (Moriarty et al. 2016). Since the main objective of the present research was to assess the impact of pregabalin on learning and memory in conditions related to DNP, this study was performed 3 weeks after STZ injection. We chose this time-point for behavioral assays to keep testing protocols in conditions as close as possible to our previous studies on the antiallodynic and antihyperalgesic properties of pregabalin in the DNP model (Sałat et al. 2013a).

Studies on the cognitive abilities of STZ-treated diabetic rodents are based on several learning tasks (Biessels et al. 2002; Moriarty et al. 2016). To assess the effect of pregabalin on learning and memory in diabetic mice, we used a passive avoidance task. This fear-motivated test is a simple and rapid cognitive task (Puzzo et al. 2014; Arias et al. 2015) which evaluates long-term memory in rodents (Lee et al. 2015). It enables the researcher to study a conditioned response to various contextual cues and, therefore, it is particularly useful to investigate emotional memory and contextual memory (Lee et al. 2015; Arias et al. 2015). Recently, a link has been found between emotional and cognitive alterations, impaired contextual fear conditioning, and diabetes-related conditions (Hwang et al. 2010; Ikeda et al. 2015; Zuloaga et al. 2015). Moreover, using the passive avoidance task, learned aspects of defensive behavior and the involvement of different brain structures in mechanisms of these types of memory (amygdala, hippocampus, respectively) can be studied (Puzzo et al. 2014; Lee et al. 2015; Arias et al. 2015). Another important reason for using the passive avoidance task was that our previous experiments on pregabalin in the DNP model were performed on albino mice, and this task is in fact one of the easiest and most readily available screens to study cognition in these mice. Albino mice tend to show significant impairments in spatial navigation tasks due to visual impairments (Puzzo et al. 2014); so, in contrast to other, more sophisticated tools which evaluate spatial navigation, learning, and memory (e.g., Morris water maze or radial-arm water maze), the passive avoidance task does not require C57BL/6J mice. In our study, in the retention phase of the passive avoidance task, we observed reduced step-through latency in the STZ-treated control group compared to non-diabetic controls, which confirmed the results of other authors showing that STZ-induced diabetes had a deleterious effect on learning and memory in numerous tasks (Biessels and Gispen 2005; Datusalia and Sharma 2014), including fear conditioning tasks (Zuloaga et al. 2015). Pregabalin did not aggravate learning deficits of STZ-treated mice as compared to STZ controls. On the other hand, pregabalin was not able to attenuate or reverse STZ-induced memory impairments. A slight reduction in step-through latency was observed in pregabalin-treated mice compared to diabetic controls, but this difference was not statistically significant.

In order to properly interpret the results from the passive avoidance task, i.e., to assess if the effects of STZ and pregabalin observed in this test were not due to altered motor skills, impaired exploratory behavior, or stress-related reactions, animals’ activity was additionally monitored by measuring ambulations, rearing, grooming bouts, and the total duration of grooming activity (Cartmell et al. 2000; Kalueff and Tuohimaa 2005). To achieve the most precise as possible insight into these activities, ambulations and rearing were measured at 3-min intervals, which strictly corresponded to the duration of a single-passive avoidance trial. Compared to non-diabetic controls, neither STZ, nor pregabalin influenced the total number of ambulations or rearing, which indicates that those treatments do not affect locomotor activity or exploratory behavior (Kalueff and Tuohimaa 2005). This is a particularly important information in reference to the observed reduced step-through latency in STZ + vehicle-treated mice and STZ + pregabalin-treated mice compared to normoglycemic controls in the retention trial of the passive avoidance task. Namely, it confirms that the effect of STZ or pregabalin on fear-motivated learning was not masked by altered locomotor activity or explorative behavior.

For a proper interpretation of the results obtained in the passive avoidance task, several other factors should also be considered. One of these is related to the anxiolytic properties of the drug under testing. In fact, the observed reduction of step-through latency in the retention trial of the passive avoidance task might be explained in terms of cognitive decline, but there is also a possibility that this effect might result from the anxiolytic-like properties of the test drug. Since pregabalin has anxiolytic properties (Toth 2014), as a next step, we decided to assess if it influences grooming behavior. In rodents, self-grooming behavior is a natural activity which is very sensitive to stress and various procedures that are made on animals. Concomitantly, it can be regarded as a spontaneous ritual behavior and a behavioral marker of animal welfare that appears in low-stress conditions (Kalueff and Tuohimaa 2005). On the other hand, under certain circumstances, grooming can also be a marker of anxiety, and anxiety can lead to increased frequency or duration of grooming. Hence, a thorough analysis of this behavior is regarded as a tool which is complementary to the available mouse tests used to study anxiety-related behavior (Kalueff and Tuohimaa 2005). For both these options (i.e., anxiolytic or anxiogenic), to differentiate between them, it is extremely important to analyze qualitative characteristics of grooming (Kalueff and Tuohimaa 2005). In our present study, STZ and pregabalin did not affect the number of grooming bouts; however, in the case of the STZ + pregabalin-treated group, a trend for a reduction of this parameter could be noted. Compared to the normoglycemic controls, the total duration of grooming activity was significantly affected by both STZ and pregabalin. Interestingly, there was also a significant difference between both STZ-treated groups. Previously, it had been reported (Kalueff and Tuohimaa 2005) that the number of bouts and, to a lesser extent, the duration of grooming may not detect anxiety, if taken alone. Hence, as a part of the analysis, we also carefully observed the qualitative features of grooming behavior. Typically, sudden bursts of rapid and very intense grooming activity with abnormal progression and incomplete and interrupted bouts were noted in STZ-treated controls but, notably, not in the non-diabetic controls. This type of high-stress-related activity seen in STZ control was almost unnoted in the STZ + pregabalin-treated group. Taken together, this might indicate that this pattern of grooming activity in STZ control was a manifestation of anxiety-related behavior, and the effect of pregabalin on its duration might be attributed to its potential anxiolytic properties. In previous studies (Kalueff and Tuohimaa 2005), anxiolytic GABAergic drugs had been shown to decrease grooming activity measures in the open field, and anxiolytic agents reduced grooming duration and frequency in rats. Summarizing, in view of the above results, this might be the main limitation of the present in vivo study, as the observed reduction of step-through latency in the retention phase of the passive avoidance task seems to be strongly influenced by anxiolytic properties of pregabalin.

The CNS plays an integral role in maintaining glucose homeostasis by promoting hormone release and through innervation of peripheral organs via sympathetic and parasympathetic actions (Ren et al. 2015). Molecular mechanisms of insulin action and its role in the brain are still not clearly understood. The presence of insulin and its receptors in the brain suggests a key role for insulin as a neuromodulator of neurotransmitter of neuronal activity (Leloup et al. 1996). Moreover, in the CNS, insulin affects feeding behavior and body energy stores, as well as various aspects of memory and cognition (Gray et al. 2014). Discrete brain areas express the insulin-responsive glucose transporter GLUT4 (Leloup et al. 1996). The levels of expression of GLUT4 protein in the cerebellum appear to respond to the level of circulating insulin (Vannucci et al. 1998). In our study, we observed significant repression of GLUT4 proteins in the pregabalin-treated diabetic mouse brains when compared to STZ-treated control animals. This suggested that pregabalin influences the expression of glucose transporters in the mouse brain.

The STZ model of diabetes has also been widely studied to understand mechanisms underlying DNP. Several etiological factors have been identified, including oxidative stress and inflammation (Gao and Zheng 2014). Also, the theory for aging and the pathogenesis of cerebral dysfunction in diabetes relates cell death to oxidative stress in strong association to inflammation and in fact NF-κB signaling (Muriach et al. 2014). The pro-inflammatory NF-κB pathway has been revealed as a key molecular system for pathologic induction of brain inflammation, which translates over-nutrition and resulting intracellular stress into central neuroendocrine and neural dysregulations of energy, glucose, and cardiovascular homeostasis (Cai and Liu 2012).

In our study, we observed significantly lower expression of COX-2, cPGES, and NF-κB p50 subunit in the brains of diabetic mice treated with pregabalin in comparison to STZ-treated control mice. Higher expression of the AhR receptor was observed in the brains of STZ + pregabalin-treated mice. Our results suggest an anti-inflammatory effect of pregabalin, both at the lower COX-2 and cPGES brain expression and also via inhibition of the NF-κB signaling by preventing p50 and p65 translocation to the nucleus.

AhR, as a nuclear factor, participates in NF-κB signaling pathways regulating inflammation (Quintana and Sherr 2013). In our study, expression of AhR was the highest and the expression of p50 was lower in the brains of pregabalin-treated mice when compared to STZ control animals. This probably suggested transrepression of NF-ĸB by AhR. The results of the study performed by Calikoglu et al. (2015) indicated anti-edematous, anti-inflammatory, and neuroprotective effects of pregabalin in an experimental head trauma model in rats. In the work of Jang et al. (2012), in a mouse model of neuropathy, pregabalin demonstrated immunomodulatory effects by inhibiting NK cell activity and splenocyte proliferation. Ha et al. (2011) indicated lower synthesis of caspase-3 and phosphorylated p38 MAPK and decreased proliferation of astrocytes in rats with spinal cord injury after administration of pregabalin. Our study confirmed these immunomodulatory actions of pregabalin in diabetic mice.

Brain inflammation may result from both acute injury and the appearance of endogenous neurotoxic metabolites associated with neurodegenerative diseases. COX-2 expression in the brain has been associated with pro-inflammatory activities, but the evidence of a direct role of COX-2 in neurodegenerative events is still controversial. The emerging role of COX-2 in behavioral and cognitive functions is still being discussed (Minghetti 2004). Prostaglandin E_2_ is among the most important mediators involved in neuroinflammatory processes (de Oliveira et al. 2008).

Neurochemical targets to reduce brain inflammation include NF-ĸB signaling, cyclooxygenase enzymes, Nrf2 transcription factors, angiotensin AT1, and sigma-1 receptors. The search for more selective drugs acting on these targets is of great interest (Jarrott and Williams 2015). The brain is very sensitive to changes in redox status; thus, maintaining redox homeostasis in the brain is critical for the prevention of oxidative damage. Nrf2 is a redox-sensitive, ligand-activated transcription factor that plays a critical role in cellular defenses against oxidative and electrophilic stress. Nrf2 activation has been shown to mitigate a number of pathologic mechanisms associated with AD, Parkinson’s disease, amyotrophic lateral sclerosis, Huntington’s disease, and multiple sclerosis (Wang et al. 2014). In our study, we observed higher expression of Nrf2 proteins in the brain of STZ-treated control mice when compared to STZ + pregabalin-treated mice. This suggested the repression of this protein and the reduction of oxidative stress in the brains of diabetic animals after pregabalin treatment.

Concluding, in this research, we have demonstrated that pregabalin does not aggravate memory deficits induced by STZ injection. Moreover, this drug has anti-inflammatory and antioxidant properties in STZ-treated mice. The results obtained in the present study might be relevant when considering the increasing use of pregabalin for epileptic and non-epileptic medical indications.

## Abbreviations

AD: Alzheimer’s disease
AhR: Aryl hydrocarbon receptor
CNS: Central nervous system
COX-2: Cyclooxygenase-2
cPGES: Cytosolic prostaglandin E synthase
DNP: Diabetic neuropathic pain
GLUT4: Glucose transporter type-4
*Ip*: Intraperitoneal
NF-κB: Nuclear factor-κB
Nrf2: Nuclear factor (erythroid-derived 2)-like 2
STZ: Streptozotocin
